# Modelling the disease: H_2_S-sensitivity and drug-resistance of triple negative breast cancer cells can be modulated by embedding in isotropic micro-environment

**DOI:** 10.1016/j.mtbio.2023.100862

**Published:** 2023-11-11

**Authors:** Silvia Buonvino, Ilaria Arciero, Eugenio Martinelli, Dror Seliktar, Sonia Melino

**Affiliations:** a Department of Chemical Sciences and Technologies, University of Rome “Tor Vergata”, via della Ricerca Scientifica, 00133, Rome, Italy; b Department of Electronic Engineering, University of Rome Tor Vergata, Rome, Italy; c Interdisciplinary Center for Advanced Studies on Lab-on -Chip and Organ-on-Chip Applications, University of Rome Tor Vergata, Rome, Italy; d Department of Biomedical Engineering, Technion Israel Institute of Technology, 3200003, Haifa, Israel; e NAST Centre, University of Rome ‘Tor Vergata’, Rome, Italy

**Keywords:** Hydrogen-sulfide, Doxorubicin, Osteocalcin, CD49b, Drug resistance, Silk fibroin, Osteoblast-like

## Abstract

Three-dimensional (3D) cell culture systems provide more physiologically relevant information, representing more accurately the actual microenvironment where cells reside in tissues. However, the differences between the tissue culture plate (TCP) and 3D culture systems in terms of tumour cell growth, proliferation, migration, differentiation and response to the treatment have not been fully elucidated. Tumoroid microspheres containing the MDA-MB 231 breast cancer cell line were prepared using either tunable PEG-fibrinogen (PFs) or tunable PEG-silk fibroin (PSFs) hydrogels, respectively named MDAPFs and MDAPSFs. The cancer cells in the tumoroids showed changes both in globular morphology and at the protein expression level. A decrease of both Histone H3 acetylation and cyclin D1 expression in all 3D systems, compared to the 2D cell culture, was detected in parallel to changes of the matrix stiffness. The effects of a glutathionylated garlic extract (GSGa), a slow H_2_S-releasing donor, were investigated on both tumoroid systems. A pro-apoptotic effect of GSGa on tumour cell growth in 2D culture was observed as opposed to a pro-proliferative effect apparent in both MDAPFs and MDAPSFs. A dedicated *ad hoc* 3D cell migration chip was designed and optimized for studying tumour cell invasion in a *gel-in-gel* configuration. An anti-cell-invasion effect of the GSGa was observed in the 2D cell culture, whereas a pro-migratory effect in both MDAPFs and MDAPSFs was observed in the 3D cell migration chip assay. An increase of cyclin D1 expression after GSGa treatment was observed in agreement with an increase of the cell invasion index. Our results suggest that the “*dimensionality*” and the stiffness of the 3D cell culture milieu can change the response to both the *gasotransmitter* H_2_S and doxorubicin due to differences in both H_2_S diffusion and changes in protein expression. Moreover, we uncovered a direct relation between the cyclin D1 expression and the stiffness of the 3D cell culture milieu, suggesting the potential causal involvement of the cyclin D1 as a bio-marker for sensitivity of the tumour cells to their matrix stiffness. Therefore, our hydrogel-based tumoroids represent a valid tunable model for studying the physically induced transdifferentiation (PiT) of cancer cells and as a more reliable and predictive *in vitro* screening platform to investigate the effects of anti-tumour drugs.

## Graphical abstract

**Figure:**
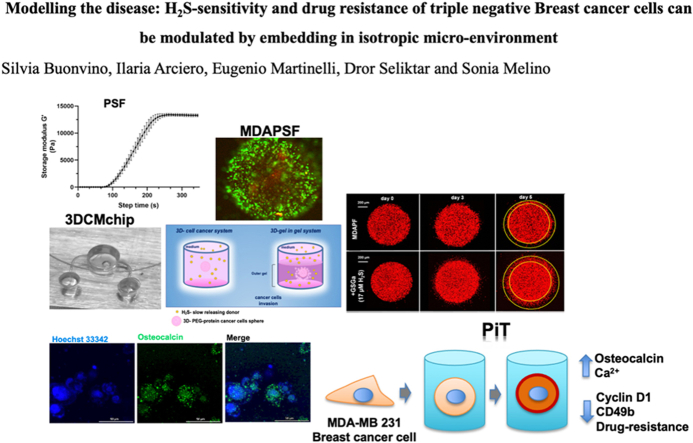

## 1 Introduction

In order to simplify the *in vivo* architecture of a tumor tissue it is possible to consider two distinct regions depending on the cellular proximity to the blood vessels. Cells that are neighboring blood vessels receive oxygen and nutrients for which they proliferate rapidly. Cells that are far away are in a condition of hypoxia and lack of nutrients [1]; therefore, these cells proliferate slowly and, for this reason, are called sleeper cells [2]. Accordingly, the tumor microenvironment *in vivo* is characterized by several factors, which are referred to as 'the Crucial P's' (inadequate perfusion, altered extracellular pH, altered vessel permeability, etc.) [[3], [4], [5]] and this makes it "metabolically hostile" for effective drug treatment. For example, due to inefficient vascularization, a drug will not reach all cells at the critical concentration required for a therapeutic effect. Therefore, some characteristics of the tumor, including drug resistance, can depend on the three-dimensional (3D) organization of cancer cells. In the last decade, several studies have highlighted relevant aspects of the tumor organization and drug response using tumor spheroids, or 3D culture systems which can better mimic the three-dimensional organization of the tumor *in vivo*. In these 3D tumor spheroids, it is possible to generate zones with different degrees of cell proliferation based on the presence of oxygen and nutrient concentration gradients [[6], [7], [8]]. In particular, tumor spheroids have three distinct zones which reflect the *in vivo* situation previously described: i) an outer layer of proliferating and metabolically active cells; ii) an innermost layer formed by quiescent cells; iii) a core made up of necrotic and/or apoptotic cells [9]. Hence, tumor spheroids can exhibit a 'hostile environment' leading to the onset of drug resistance, similar to that which is observed *in vivo* [10]. Furthermore, the tumor spheroids are also resistant to various physical stimuli, including heat, radiation and photodynamic therapy [11]. Therefore, spheroidal systems simulate conditions *in vivo* much more closely than 2D systems. The characteristics described above, together with the simplicity of producing the tumor spheroidal model and possibility to apply it to high-throughput screening, makes the spheroid model suitable for new drug screening assays [9,12]. The biomimetic aspects of 3D tumoroids are also exploited to study dynamic processes such as the growth and invasiveness of some solid tumors. In general, 3D cell growth systems give the possibility to greatly expand knowledge on the effects of the microenvironment on the invasiveness of cancer cells and their response to mechano-physical stimuli and drugs. However, many studies are still needed in order to understand the growth-related gene expression variations in 3D tumor cell systems, and to optimize and make tunable the 3D culture systems for mimicking the physiological environment. Simple tumor spheroids obtained through commercial *kits* are limited because they do not provide the ability to control cell density or mechanical properties of the spheroid. Recently, a valid alternative to commercial tumor spheroids kits has been proposed using photopolymerizable semi-synthetic hydrogels. The hydrogels are used to make tumor cell microspheres with control over the chemical and mechano-physical properties of the system. In addition, these hydrogel microspheres can be loaded with different cell types and at different cell densities to precisely control the cellularity of the system. Thus, the hydrogel-based tumoroids allow to assess different conditions and effects on tumor cell proliferation, cell invasiveness, as well as on the expression of genes of metabolic pathways relevant to tumorigenicity and drug resistance.

In the present study we applied PEG-protein hydrogels in order to produce cancer cells 3D microspheres with tunable mechanical properties and cellular density. Two different photopolymerizable hydrogel systems were used to produce the microspheres: PEG-fibrinogen [13] and PEG-silk fibroin [14]. The cell proliferation and migration of the human breast cancer MDA-MB 231 cells growing in the 3D hydrogel tumoroids were analyzed. The MDA-MB 231 cell line is reported as a subtype triple negative [15], based on conventional classification of increasing order of aggressiveness. Our experiments focused on growth of the cells with and without H_2_S-releasing donors and comparing this growth kinetic to the conventional 2D cell culture systems. The anticancer activity of H_2_S slow-release donors is well documented in 2D cell culture systems and herein we sought to expand upon this knowledge using 3D culture experiments. In both colon and breast cancer, GYY4137 reduced tumor cell proliferation by arresting the cell cycle and inducing apoptosis [16,17]. Diallyl disulfide (DADS) and diallyl trisulfide (DATS), H_2_S-releasing organosulfur compounds (OSCs) derived from garlic, have also been shown to reduce the viability and migration of breast cancer cells [[18], [19], [20], [21]]. In general, H_2_S donors exert antitumor activity by acting on the EGFR/ERK/MMP-2, PTEN/Akt, PI3K/Akt/mTOR pathways and by inhibiting NF-kB [22], whose excessive activation is linked to the progression of ER-negative tumors [23,24]. NF-kB can regulate the expression of matrix metalloproteases, proteins whose overexpression in tumors is linked to invasiveness and the ability to form metastases. Through the action on NF-kB, these slow-release H_2_S donors can therefore decrease the expression of metalloproteases and the tumor cell migration. This effect was observed by treating the breast cancer cell line MDA-MB 231 with the donor DATS: a reduction in mRNA expression and enzymatic activity of the metalloproteases MMP-2 and MMP-9 was observed [18]. A decrease in MMP-9 expression and activity was also found when treating MDA-MB 231 cells with DADS, which was able to inhibit the metastatic potential of this cell line [25]. In addition, DADS also decreased the viability of cancer cells by increasing the activity of caspases 3 and 9, essential proteins in the apoptotic process, by increasing the expression of the pro-apoptotic factor Bax and decreasing the expression of Bcl-2, which is an anti-apoptotic protein. Additional evidence of the anticancer activity of H_2_S slow-release donors was obtained with *in vivo* experiments using tumor cell xenografts. Treatment with DADS reduced the weight and volume of tumors obtained by implanting MDA-MB 231 cells in a naked mouse [25,26]. Accordingly, these compounds, alone or in combination with other anticancer drugs, are potential candidates for anti-cancer therapies, even if the anticancer mechanisms underlying their action still need further clarification. The effect of H_2_S depends on its concentration, that is positive with anti-inflammatory and antioxidant effects at low concentrations and, on the contrary, is cytotoxic at high concentrations. Further, the molecular characteristics of the donor, the rate of H_2_S release and the cell lines used are very important aspects to consider for evaluating the efficacy of a H_2_S-donor anti-cancer therapy [22]. Our previous studies suggested the possibility to use glutathionylated OSCs derived from garlic as slow-release donors of H_2_S [19]. In particular, we have produced GSGa, a water-soluble garlic extract, characterized by the presence of conjugated glutathionyl-OSCs and capable of slowly release H_2_S. Although GSGa has pro-apoptotic properties on tumor cells [19], this H_2_S-donor has been found to stimulate the proliferation and migration of human cardiac mesenchymal stem cells (Lin^−^Sca-1^+^ hMSCs) [20,27]. In this study, we have analyzed the effects of GSGa on the 3D MDA-MB 231 culture systems produced using PF and PSF hydrogels cancer cells microspheres. In these 3D systems the effects on the cellular viability and migration/invasiveness were opposite with respect to the observations made in the 2D cultures. We found a reverse relationship between the cyclin D1 expression and the stiffness of the 3D cancer cell system, and this expression was also associated to variation of the histone H3 acetylation. Moreover, the rheological change of the tumoroid micro-environment (TME) leads also to a *trans*-differentiation of the breast cancer cells inducing an osteoblast-like behavior and reducing the glycoprotein P (Pgp) expression. Consequently, a major sensitivity of these cells to anticancer agents, such as doxorubicin, which is different from H_2_S-donors, was observed.

## 2 Materials and methods

### 2.1 Hydrogels and tumoroids preparations

**Fig. 1:**
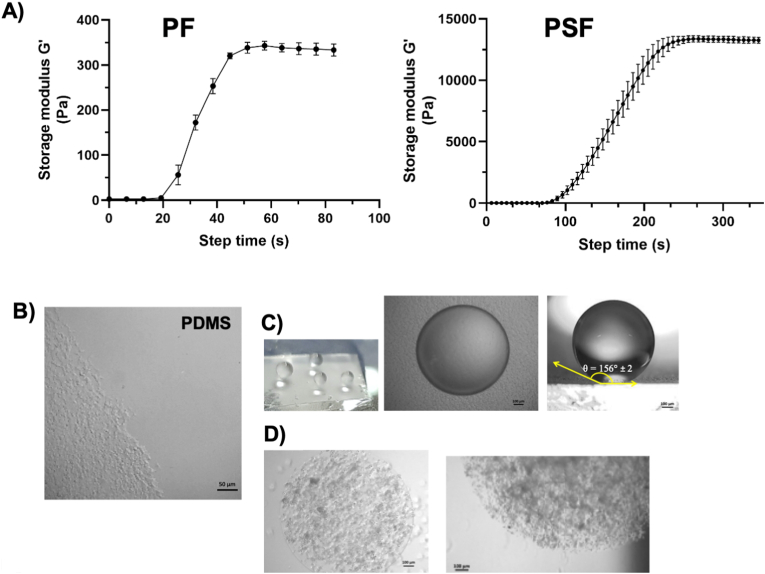
Rheology of the hydrogel and MDA-MB 231 cells microspheres production. A) Rheologic measurements of shear storage modulus, G′ for a) PF and b) PSF hydrogels; **B)** micrographs of the PDMS superhydrophobic surface; **C**) digital images of the microspheres on the superhydrophobic surface, micrographs of the 3 μl PF-spheres on the nanostructured superhydrophobic surface and the contact angle with the surface of a water drop (3 μl) (θ ≥ 150°); **D)** micrographs of MDAPF and MDAPSF microspheres in cell culture medium. Scale bars are of 100 μm.

PEG-fibrinogen was obtained using the protocol previously optimized by Seliktar et al. [13] and the PEG-silk fibroin was produced by purification and PEGylation of the silk fibroin from *Bombix mori* cocoon, as previously described by Melino et al. [14]. The silk fibroin purification is shown in Fig. 1S (see Supplementary Materials). The PEG-fibrinogen (PF) and PEG-silk fibroin (PSF) hydrogels were obtained using the PF (62.6 μl of PF 13 mg/ml fibrinogen, 1.6 μl of 30 % w/v PEG-diacrylate (PEGDa 10 kDa), 1 % w/v of Irgacure 2959 and PBS for a total volume of 100 μl) and the PSF precursor solution (70 μl of PSF 6 mg/ml fibroin, 30 μl of 30 % w/v PEGDa 10 kDa, 1 % w/v of Irgacure 2959 for a total volume of 100 μl), respectively.

A superhydrophobic surface of polydimethylsiloxane (PDMS) was fabricated using the procedure described by Patent LDO0252 (Film super idrofobico - Mesap IPC – B08B17/06 B29C39/14 |B29C39/148) [29] and characterized by optical microscopy at a magnification of 100×. MDA-MB 231 cancer cells at a density of 10^4^ cells/μl were suspended in the precursor solutions and put 3 μl on a superhydrophobic surface using a micropipette. The drops were photopolymerized immediately by exposure under UV (365 nm, 5 mW/cm^2^) for 2 min.

### 2.2 Rheometric analysis of the hydrogels

The mechanical properties of the hydrogels were estimated by strain-rate controlled oscillatory shear rheometry using a TA Instruments AR-G2 rheometer (ARES, TA Instruments, New Castle, DE, USA). The rheometer was equipped with a UV curing cell (365 nm, 5 mW/cm^2^) placed underneath the parallel-plate geometry. The shear storage modulus (G′) of the hydrogels was determined using a 20 mm parallel-plate geometry by applying a sinusoidal shear deformation to the sample liquid. Dynamic time sweeps were performed at 25 °C, 2 % sinusoidal strain, and 3 rad/s constant frequency, continuously monitoring the rheology of the materials during the photo-polymerization (*in situ* rheometry) using the Advantage Instruments Control AR Software. A sample (200 μl) of the liquid precursor was pipetted on the lower plate, and the upper plate was lowered until it reached a proper gap (∼600 μm). The UV light was turned on after 30 s of rheological measurements. The measurement was carried out for ∼5 min, or until the maximum value of G’ was reached. A gas flow-cover was used to reduce UV radiation.

### 2.3 Garlic water-soluble extract production from *Allium sativum* L

The garlic water-soluble extract was produced as previously described [19,27]. Briefly, 5 g of garlic cloves were crushed in liquid N_2_ for about 10 min in the presence of 50 mM Tris-HCl buffer, pH 7.5 with 100 mM reduced glutathione (GSH). After centrifugation the water-soluble fraction was stored at −20 °C for molecular characterization with RP-HPLC. This analysis was performed using mod. LC-10AVP (Shimadzu, Milan, Italy), equipped with a UV detector (Shimadzu, Milan, Italy) and a C_18_ column (150 mm × 4.6 mm, 5 μm, CPS Analitica, Rome, Italy), using 0.1 % trifluoracetic acid as solvent A and 80 % CH_3_CN, 0.1 % trifluoracetic acid as solvent B and with a solvent B gradient (0–5 min, 0 %; 5–55 min, 60 %; 55–60 min, 60 % and 65–85 min 90 %). Elute was monitored at 220 nm. The dry weight of the GSGa extract obtained by lyophilization was used to assess its concentration in mg/ml.

### 2.4 H_2_S release assay

The methylene blue (MB) assay was used in order to evaluate the H_2_S production and release by GSGa, GYY4137 and Na_2_S, as previously described [19,20,27]. Briefly, each sample with 1 mM dithiothreitol (DTT) in 50 mM Tris HCl, pH 7.4 (150 μl final volume) was incubated at 37 °C on a shaker for 30 min. Then, 20 μl of solution I (20 mM N′, N′-dimethyl-p-phenylene-diamine-dihydrochloride in 7.2 M HCl) and 20 μl of solution II (30 mM FeCl_3_ in 1.2 M HCl) were added after the incubation to each solution and after gentle mixing of the solutions for 10 min at room temperature the absorbance was measured at 670 nm. Na_2_S was used to elaborate a standard curve (Fig. 2S in Supplementary Materials) and the H_2_S- release from GYY4137 was also tested by the MB assay and compared to that from GSGa.

**Fig. 2:**
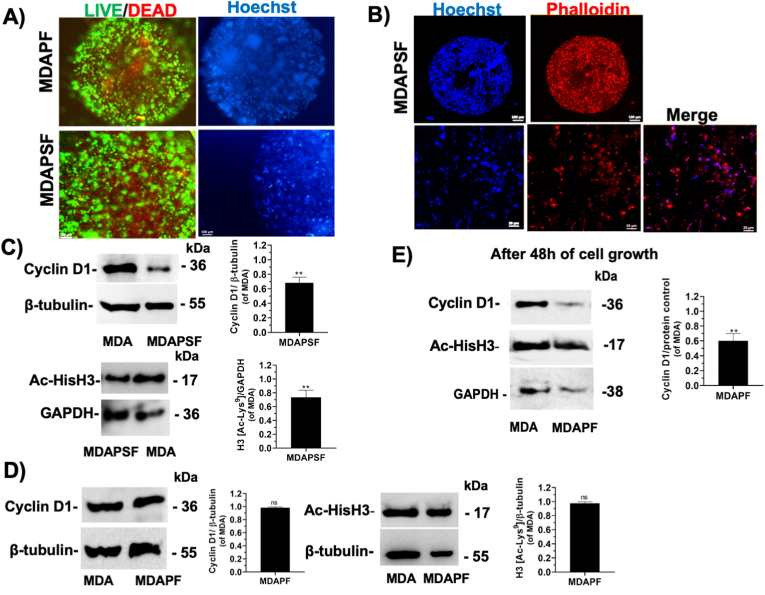
Cell viability of MDAPF and MDAPSF microspheres and cyclin D1 and Ac-Hist H3 expression of the different cell growth systems. A) (a) L/D cell viability assay of MDAPFs (on the left) and a live-cell fluorescent staining with Hoechst 33342 (on the right) after 3 and 1 days of cell growth, respectively, (b) L/D assay of MDAPSFs and using a live-cell fluorescent staining of the nuclei with Hoechst 33342 after 3 days and 2 weeks of cell growth, respectively; **B)** confocal micrographs of cells in the MDAPSF system after 2 weeks of cell growth, the nuclei (in blue) were stained using a live-cell fluorescent staining with Hoechst 33342 and the cell cytoskeleton (in red) was stained using Phalloidin AlexaFluor-660. Expression of cyclin D1 and of Ac-Hist H3 in MDAPSF **C)** and MDAPF **D)** after 24 h of cell growth and **E)** in MDAPF microspheres after 48 h of cell growth. Scale bars are of 100 μm in **A)** and 100 and 20 μm in **B)**. **p value ≤ 0.01.

### 2.5 Cell cultures and cell viability assay

Tumour cells of the MDA-MB 231 cell line were cultured in Dulbecco's Modified Eagle Medium (DMEM) (Gibco, Italy), containing 10 % of Fetal Bovine Serum (FBS) (v/v) (Gibco, Italy), 1 % of penicillin-streptomycin (w/v) (Sigma-Aldrich, Italy) and 2 mM l-Glutamine solution (Gibco, Italy). The treatments of cancer cells in 2D cultures were performed adding 100 μM of GYY4137, or 2 μM of doxorubicin (Sigma-Aldrich, Italy) or GSGa, at concentrations releasing 4.0, 17 and 34 μM of H_2_S, after cell adhesion on TCP in the cell culture medium. The cell viability was then assessed after 24 h of treatment. The treatments of tumoroids were performed adding GSGa at concentrations releasing 17 and 34 μM of H_2_S or 2 μM of doxorubicin (Sigma-Aldrich, Italy) in the cell culture medium after 24 h of cell growth. The cell viability was then assessed after 24, 48 or 72 h depending on the specific treatment. Cell viability was tested either by WST-1(4-[3-(4-lodophenyl)-2-(4-nitrophenyl)-2H-5-tetrazolium]-1,3-benzene disulfonate (Cell Proliferation Reagent WST-1, Roche, Mannheim, Germany) or WST-8 assay (2-(2-methoxy-4-nitrophenyl)-3-(4-nitrophenyl)-5-(2,4-disulfophenyl)-2H-tetrazolium, monosodium salt) (Cell Counting Kit 8 (WST-8 /CCK8) (ab228554), Abcam) [30,31], as indicated in the figure legends. After each treatment, the medium was replaced with fresh DMEM high glucose without phenol-red (GIBCO, Italy) containing tetrazolium salt WST-1 or WST-8 (5 % v/v). The cells or tumoroids were incubated for 3 h at 37 °C, 5 % CO_2_ and the absorbance of the medium was evaluated using a microplate reader at a wavelength of 450 nm for WST-1 assay and of 460 nm for WST-8 assay. The viability of MDA-MB 231 cells embedded in 3D microspheres was also monitored by fluorescence microscopy using a LIVE/DEAD® Cell Imaging Kit (L/D assay) (488/570) (Molecular Probes, Life Technologies, Thermo Fisher Scientific, Milan, Italy) and using a Zeiss fluorescence microscope Axio Observer 7 Zeiss. Alizarin Red S (Sigma-Aldrich, Italy) staining of the MDAPSFs for the identification of the calcium rich deposits was performed fixing the spheres with 4 % PFA and staining with Alizarin red solution, pH 4.1, for 10 min and washing with H_2_O_dd_, according to the specific protocol. The staining was assessed by optical microscopy using a Zeiss microscope (Primovert, Zeiss, Italy).

### 2.6 Scratch wound healing assay

MDA-MB 231 cells were seeded into 24-well plates (6.5 × 10^4^ cells/cm^2^) and incubated over night at 37 °C with 5 % CO_2_ to reach confluency the day after. After 24 h, a scratch-wound was created with a 1 ml sterile pipette on the cell monolayer of each well. The medium was then removed and cells washed with PBS and fresh medium was added to each well (800 μl/well). Area of the scratch-wound at time 0 and after 24 h was measured with ImageJ Software. Percentage of wound closure was measured as follows:

(1) Wound closure (%) = (Wound surface area after 24h / Wound surface area at time 0) x 100

### 2.7 3D cell migration chip and gel in gel assay

The 3D cell migration chip (3DCM-chip), that was described in detail in Italian Patent No. 102021000025460 [32], was used for performing the *gel in gel* assay [33]. Briefly the *gel in gel* assay is based on a hydrogel microsphere (inner gel), in which cells are embedded, incorporated in an outer gel with lower stiffness, where cells are able to migrate. The cell migration index was calculated measuring the size of the circular crown around the cell sphere [33] using the following equation:

(2) $$\text{migration}\;\text{index}=\frac{\pi *{{(R}_{\text{av}e\text{rage}}^{2}‐r_{\text{av}e\text{rage}}^{2})}^{\text{sample}}}{\pi *{{(R}_{\text{av}e\text{rage}}^{2}‐r_{\text{av}e\text{rage}}^{2})}^{\text{control}}}$$

where R is the average of final radius and r is the average of radius at time 0.

Cell migration studies were performed using MDA-MB 231 or MDA-MB 231/mCherry cancer cell lines (Lonza, Basel, Switzerland) at cell density of 10^4^ cells/μl and 4 × 10^4^ cells/μl, respectively. MDA-MB 231/mCherry were used at cell density of 4 × 10^4^ cells/μl in order to speed-up the cell migration/invasion in the outer gel for technical requirements in the time-lapse analysis by confocal microscopy.

### 2.8 Fluorescence microscopy of MDAPFs and MDAPSFs

MDA-MB 231 cells were cultured in Dulbecco's Modified Eagle Medium (DMEM) (Gibco, Italy), containing 10 % of Fetal Bovine Serum (FBS) (v/v) (Gibco, Italy), 1 % of penicillin-streptomycin (w/v) (Sigma-Aldrich, Italy), 2 mM l-Glutamine solution (Gibco, Italy) and embedded into the PF or PSF mixture. Immunofluorescence on MDAPF and MDAPSF cultures was performed using a live-cell fluorescent staining of the nuclei with Hoechst 33342 (Sigma-Aldrich, Italy) for 4 h and then the cells were fixed with 4 % paraformaldehyde (PFA) in PBS for 30 min at room temperature, permeabilized with 0.3 % Triton X-100 for 5 min and maintained in a blocking buffer (10 % of FBS (v/v), 0.1 % of Triton X- 100 (v/v) and 1 % of glycine (w/v) in PBS) overnight at 4 °C. MDAPSFs were then incubated for 2 h at room temperature with 1:200 v/v Phalloidin conjugated AlexaFluor 660® (Thermo Fisher Scientific, Invitrogen, USA), in PBS with 1 % albumin with 20 mM Gly solution or overnight with primary antibody for osteocalcin (Thermo Fisher Scientific, Invitrogen, USA) and CD49b (Lyoplate BD Lyoplate™ Human Cell Surface Marker Screening Panel, BD Bioscience, Canada) followed by the appropriate Alexa fluorochrome-conjugated secondary antibody (488 nm) (Thermo Fisher Scientific, Invitrogen, USA). Confocal microscopy was performed using a Stellaris Leica microscope platform.

### 2.9 Protein expression by western blot analysis

Proteins were extracted from MDA-MB 231 cells, MDAPF and MDAPSF using 100 μl of sample buffer for SDS-PAGE. Samples were vortex and boiled for 5 min and centrifuged for 5 min at 10000 rpm and stored at −20 °C. The cell extracts were electrophoresed on 10 or 15 % polyacrylamide gel and electro-blotted on PVDF membrane (Sigma-Aldrich, Italy). The membranes were then blocked and probed overnight at 4 °C with primary monoclonal antibodies: Ab-AcH3 rabbit, Ab-ERK1/2 rabbit, Ab-p-ERK1/2 rabbit (Sigma-Aldrich, Italy), Ab-cyclinD1 rabbit (Cell Signaling Technology, USA), Ab-Pgp rabbit (Abcam, Cambridge, UK), Ab-Osteocalcin mouse (Thermo Fisher Scientific, Invitrogen, USA). Immunoblots were next incubated with the secondary antibodies (dilution 1:3000) (Cell Signaling Technology, USA) for 4 h at room temperature. Immunoblot with Ab-β-actin rabbit, Ab-GAPDH rabbit or Ab-β-tubulin mouse (Sigma-Aldrich, Milan, Italy) were also probed for controlling the protein loading. Immunoblots were probed with a Super Signal West Pico kit (Thermo Scientific, USA) to visualize signal, followed by exposure to a Fluorchem Imaging system (Alpha Innotech Corporation-Analitica De Mori, Milan, Italy).

### 2.10 Statistical analysis

GraphPad Software (GraphPad Prism version 5.0, San Diego, CA, USA) was used for performing the statistical analysis of the data from three or five independent experiments and were quantified and analyzed for each variable using a one-tailed Student's t-test or one-way ANOVA test. A *p* value < of 0.05 was considered to be statistically significant. Standard deviations were calculated and presented for each experiment.

## 3 Results

### 3.1 MDAPF and MDAPSF microspheres production

PEG-fibrinogen hydrogels (PFs) and PEG-silk fibroin hydrogels (PSFs) were obtained according to previous studies [13,28,34,35]. The viscoelastic properties of the PFs and PSFs were assessed using oscillatory shear rheometry. Significant differences in the shear storage moduli (G′) were observed between hydrogels, as shown in Fig. 1A. PFs with 0.5 % w/v PEGDa (10 kDa) without embedded cells was characterized by a shear storage modulus of G’ = 0.3 kPa, while the PSFs with 9 % w/v PEGDa (10 kDa) was characterized by a shear storage modulus of G’ = 13.4 kPa. This last stiffness is comparable to the *in vivo* tumor tissue of invasive ductal carcinoma that was observed to be 6–10 kPa [36], the most common type of invasive breast cancer [37]. The superhydrophobic surface of PDMS (Fig. 1B) was fabricated with nano-structures using the procedure described by Patent LDO0252 [29, Film super idrofobico - Mesap IPC – B08B17/06; B29C39/14 |B29C39/148] and it was characterized by microscopy and the analysis of the contact angle. The contact angle on the PDMS surface was equal to 156° ± 2, as shown in Fig. 1C. Spheres of MDA-MB 231 cancer cells in their respective PF and PSF hydrogel matrices (MDAPFs and MDAPSFs) (Fig. 1D) were produced using photopolymerization with UVA of drops of 2 or 3 μl of PF or PSF precursor mixture with cells inside, placed on the superhydrophobic nano-structured PDMS surface.

The cell viability of the MDA-MB 231 cells in the microspheres was assessed by LIVE/DEAD (L/D) assay and the dead cells, visible as red dots, were mainly present in the central area of them (see Fig. 2A). This finding suggests that a core is formed in the 3D system composed of necrotic and/or apoptotic cells, probably due to the reduced permeability of oxygen and nutrients within the sphere. The central zone, characterized by less viable cells, typically forms in spheroidal systems with a radius greater than about 200 μm, as is the case of the PF spheroids used in these experiments (diameter ≥600 μm), and reflects an important pathophysiological aspect of real tumor masses [38,39]. In tumors, zones with different degrees of cell proliferation have been observed due to the presence of oxygen and nutrient concentration gradients [40]. Thus, this is one aspect of native tumors that the MDAPFs and MDAPSFs spheroidal systems can efficiently simulate as 3D tumor cellular models.

Although the internal part of the spheres was characterized by the presence of dead cells (red cells) the observed prevalence of the green fluorescence, in both cases, indicated that a large proportion of the cells were growing under healthy conditions. The good cell viability of the MDA-MB 231 cells in both the hydrogel spheres was also observed by a live-cell fluorescent staining of the nuclei with Hoechst 33342 (see Fig. 2A and B) using fluorescence microscopy. MDAPSF spheres were observed by confocal microscopy after 2 weeks of cell growth, staining nuclei with Hoechst and the cytoskeleton with Alexa-Fluor phalloidin (Fig. 2 B). The non-elongated morphology of the cells was probably due to the PSF hydrogel stiffness.

### 3.2 2D and 3D cell culture systems show different expression of cyclin D1 and Ac-Hist H3

The expression of cyclin D1 depends on the biophysical and biochemical characteristics of the microenvironment around the cell through the integrin-mediated signaling pathway, involving the Rho and Rac proteins [41] and mediated by Frizzled receptors, *i.e*. the canonical Wnt/β-Catenin pathway [42]. Moreover, also the acetylation of the Histone H3 (Ac-Hist H3) has been correlated to physical stimuli effects [43]. Histone acetylation is a post-translational modification that plays an important role in cell cycle regulation, cell proliferation and differentiation, regulating the chromatin accessibility to transcription factors [44].

Therefore, here the cyclin D1 and Ac-Hist H3 expression in dependence of the stiffness of the microenvironments were investigated. A significant down-regulation of the cyclin D1 and a reduced acetylation of the Histone H3 in MDAPSFs were observed after 24 h, as shown in Fig. 2C. The decrease of the cyclin D1 expression can be related to the decrease of the cell proliferation and migration in the 3D system with respect to the 2D cell culture. Cyclin D1 down-regulation was also observed in the MDAPF microspheres, but only after 48 h of cell growth (Fig. 2C and D), indicating that the lower stiffness induces the changes in the expression only at longer times.

The effect on the histone H3 acetylation observed in MDAPSFs, at higher stiffness, was in agreement with the reduced histone acetylation of both the histones H3 and H4 observed in human mammary epithelial cells cultured in 3D system respect to 2D cultures [45].

Nuclear mechano-sensing directly inﬂuences chromatin organization, epigenetic modiﬁcations and gene expression. Actomyosin contractile forces, in response to matrix stiffness cues, are transferred to the nucleus to alter expression of mechanosensitive genes [46]. Recently, it has been demonstrated that matrix stiffness can alter lamina-associated chromatin, genome-wide chromatin accessibility through differential HDAC activity, and even DNA methylation of the YAP promoter [46,47]. Taken together, these results indicate that the epigenome is affected by the cellular microenvironment and that hydrogel cell culture platforms can be rationally designed to reproduce physiological conditions that favour a desired cellular activity.

### 3.3 Effects of GSGa treatment of MDA-MB 231 in 2D and 3D culture systems

The role of three-dimensionality in the response of MDA-MB 231 cells to the GSGa treatment was also studied. GSGa is a specific garlic extract able to slowly release H_2_S [19]. In our previous studies, GSGa has been shown to induce apoptosis in 2D cultured human lymphoma cells [19], but, conversely, it was also able to stimulate in 2D the proliferation and migration in human cardiac mesenchymal stem cells (human Lin^−^Sca-1^+^ cMSC) [27,35].

**Fig. 3:**
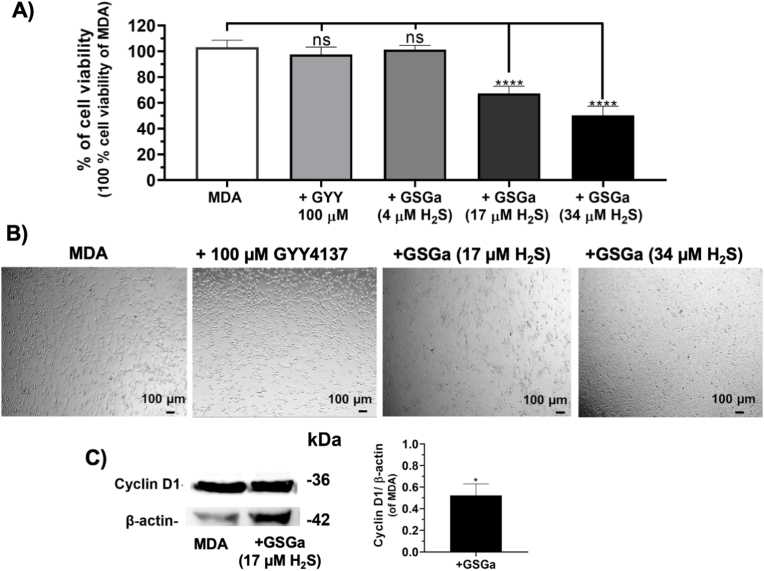
Effects of the H_2_S- donor GSGa on the MDA-MB 231 cancer cell line in 2D cell cultures. A) Cell viability WST-1 assay of MDA cells treated with the H_2_S slow-releasing donors: GYY4137 (100 μM), GSGa releasing 4.0, 17.0 and 34.0 μM of H_2_S after 24 h of treatment; **B)** optical micrographs of the cancer cells after 24 h of treatment; **C)** expression of cyclin D1 assessed by western blot analysis after 24 h of cell growth. The results were obtained by three biological replicas. Error bar indicates S.D. *p value ≤ 0.05, ****p value ≤ 0.0001.

Firstly, the effect of H_2_S donor GSGa on MDA-MB 231 cell viability in 2D cultures was evaluated using increasing concentrations of GSGa releasing 4.0, 17.0 and 34.0 μM of H_2_S, as shown in Fig. 3. The results show a cell viability decrease in concentration dependent manner of the concentration of H_2_S-releasing in the cell culture medium. In particular, after 24 h of treatment with 17.0 μM of H_2_S, a 33 % decrease in cell viability was observed. The effect of GSGa was also compared to that of GYY4137, another slow-releasing H_2_S donor that is known for its ability to reduce tumor cell proliferation [17]. MDA-MB 231 cells were treated at different concentrations of GYY4137 (50 μM, 100 μM, 300 μM) for 24 h (data not shown). No significant decrease of the cell viability of the cancer cells after treatment with 100 μM of GYY4137 was observed, according to a lower effect with respect to GSGa at the concentration releasing 17.0 μM of H_2_S (Fig. 3A and B). This antiproliferative effect of GSGa on the MDA-MB 231 tumor cell line was comparable to the effects of H_2_S donors previously obtained with other cancer cell lines, such as HuT 78 and MCF 7 cancer cell lines [19,20]. The anti-proliferative effect on the MDA-MB 231 cancer cell line in 2D cultures was also related to a down-regulation of cyclin D1 expression in these cancer cells treated with GSGa (at the concentration releasing 17.0 μM of H_2_S), as shown in Fig. 3C.

**Fig. 4:**
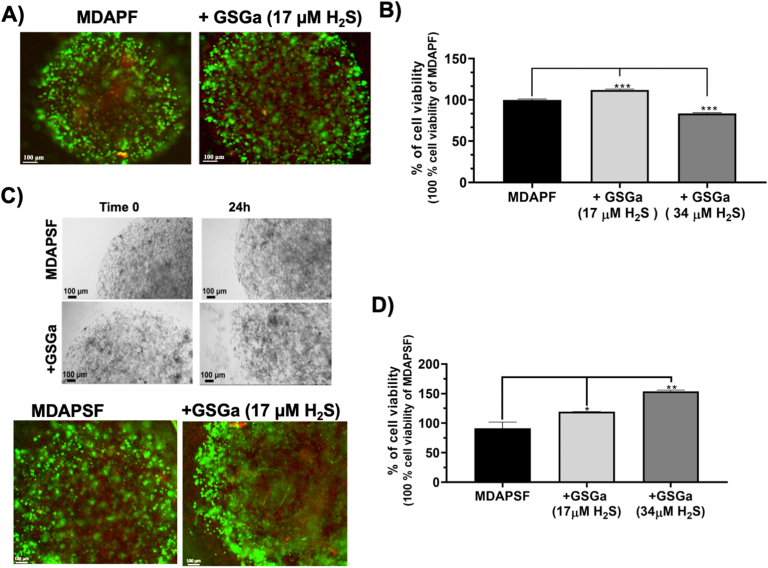
Effects of GSGa treatment on the cell viability of 3D MDAPF and MDAPSF microspheres. A) L/D cell viability assay performed on MDA embedded in MDAPF microspheres (3 μl of volume) after 1 day of cell growth in the absence or in the presence of GSGa (17.0 μM of H_2_S); **B)** WST-8 cell viability assay performed on MDAPFs after 2 days of cell growth without and with GSGa (releasing 17.0 μM or 34.0 μM of H_2_S); **C)** optical micrographs and L/D assay of MDAPSFs (3 μl of volume) after 1 and 3 days, respectively, of cell growth without and with GSGa (17.0 μM of H_2_S) in the cell culture medium; **D)** cell viability WST-1 assay of the MDAPSFs after treatment of 24 h with different concentrations of GSGa (releasing 0, 17 and 34.0 μM of H_2_S). Scale bars are of 100 μm. The results were obtained by three biological replicas. Error bar indicates S.D. *p value ≤ 0.05, **p value ≤ 0.01, ***p value ≤ 0.0005.

The effects of GSGa on the 3D cell culture systems were assessed using both MDAPFs and MDAPSFs. No cytotoxic effects of GSGa on both 3D culture systems were observed using the same concentration of GSGa (680 μg/mL GSGa releasing 17.0 μM of H_2_S) that was cytotoxic for the 2D cell cultures. In Fig. 4A and C the cell viabilities of the MDAPFs and MDAPSFs by L/D assays in the presence of GSGa in the medium are shown. A high viability of the cancer cells was observed in the presence of GSGa releasing 17.0 μM of H_2_S that was comparable to the control. The cell viability by WST-1 assay was also assessed for a quantitative evaluation of the effect (Fig. 4B and D), demonstrating that the cell viability of cancer cells in the MDAPSFs was not affected, also at a higher concentration of GSGa (releasing 34.0 μM of H_2_S), as observed in the case of MDAPFs, and on the contrary it was increased.

The effects of the *gastransmitter* H_2_S on these types of 3D cancer cell systems were never tested before. These results could be explained by the different diffusion gradients of fluids in 3D systems due to the different stiffness of the PF and PSF hydrogels and to the different “*dimensionality*” of the cell culture system used.

### 3.4 Effects of GSGa treatment on cancer cell invasion in 3D systems

**Fig. 5:**
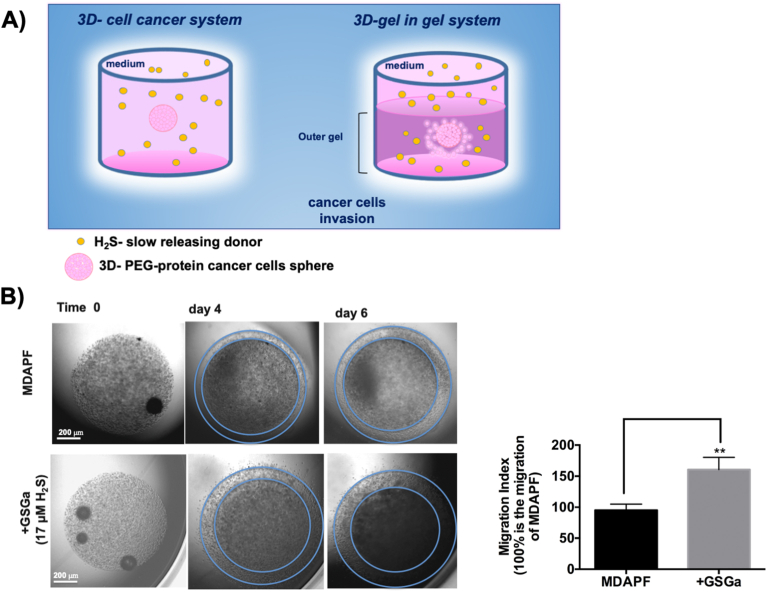
**Tumor cell invasion assay. A)** Schematic representation of the “*gel in gel*” 3D system and the H_2_S-donor treatment; **B)** optical micrographs of the **“***gel in gel*” cell invasion assay of MDAPF (3 μl, 10^4^ cells/μl) monitored for 6 days of cell growth in the absence or in the presence of GSGa (17.0 μM H_2_S released) in the cell culture medium. The cell migration index was calculated by the crown indicated in the figure. **p value ≤ 0.01.

More information on the effect of GSGa in 3D cultures was obtained using the *"gel in gel*" migration assay (Fig. 5A). This *in vitro* assay can be used for simulating the invasive behavior of a tumor mass [33]. MDAPFs/MDAPSFs were used as inner gel and after 24 h were embedded into the outer gel by photopolymerization. The outer gel was made of PF hydrogel with fibronectin and without the addition of PEGDa, it has been optimized in terms of chemical composition and mechanical properties for the massive migration of MDA-MB 231, as previously described [33].

Cell migration from the inner to the outer gel was followed by optical microscopy for six days and the size of the crown formed in the external gel, due to cell invasion (migration and proliferation), was compared between the samples. The same concentration of GSGa (releasing 17.0 μM of H_2_S) that in 2D MDA culture induced a decrease in cell viability, in 3D culture stimulated the cell migration. The size of the crown formed around the outer gel was of ∼50 % greater for the samples grown in the presence of GSGa in the culture medium rather than in the absence of GSGa.

**Fig. 6:**
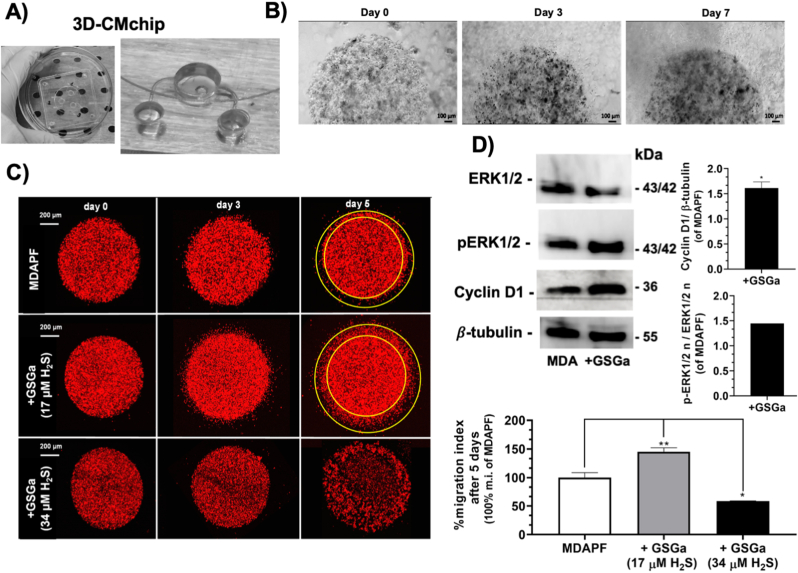
Effects of GSGa treatment on cell migration of MDAPFs. A) Digital image of the 3D cell migration chip (3DCM-chip) (Italian Patent No. 102021000025460) [32]; **B)** micrographs of the MDAPFs (3 μl, 10^4^ cells/μl) after 0, 3 and 7 days of cell growth in the 3DCM-chip where is observable the cell invasion in the outer gel; **C)** confocal micrographs of invasion assay of MDAmCherryPFs (3 μl, 4 × 10^4^ cells/μl) in the 3DCM-chip monitored for 5 days of growth in the absence and in the presence of GSGa releasing 17.0 μM H_2_S and 34.0 μM H_2_S and migration index percentage; **D)** ERK 1/2, p-ERK 1/2 and cyclin D1 expression in MDAPFs in the absence and in the presence of GSGa releasing 17.0 μM H_2_S after 24 h of cell growth in 3D. All the results were obtained by three or four biological replicas. Error bar indicates S.D. *p value ≤ 0.05, **p value ≤ 0.01.

In order to perform this cell invasion assay with a more simple, handy and reliable system, we have designed and fabricated a “*lab on a chip*” named “3D cell migration chip” (3DCM-chip) (Italian Patent No. 102021000025460) [32] (Fig. 6A). In Fig. 6 B we show the optical micrographs of the MDAPFs in the 3DCM-chip, with the cell invasion into the outer gel visible over time.

The latter system was used for analyzing the cell invasion of MDAmCherryPFs at different concentrations of GSGa (releasing 0, 17.0 and 34.0 μM of H_2_S) in the cell culture medium (see Fig. 6C). In agreement with the results obtained with the classical “*gel in gel*” assay, the presence of GSGa at a concentration releasing 17.0 μM of H_2_S in the culture medium increased the invasiveness of the cancer cells of ∼50 %, and at a higher concentration releasing 34.0 μM of H_2_S induced the cell death of the MDAPFs (Fig. 6C), in agreement to the cell viability shown above in Fig. 4B.

The expression of cyclin D1 and the ERK1/2 activation in the MDAPFs were evaluated after treatment with GSGa by western blotting (Fig. 6D). ERK pathway is normally associated with enhanced cell proliferation. ERK1/2 activated by phosphorylation translocate into nucleus where they activate the transcription of several genes and participate in the regulation of G_1_-to S-phase transition [48]. The activation of ERK1/2 is positively related to cyclin D1 induction and cell cycle progression [[49], [50], [51], [52], [53]] and the expression of cyclin D1 in early G1 phase of the cell cycle has been demonstrated to depend on sustained ERK1/2 activation in several studies [54].

After 24 h of cell growth in the presence and in the absence of H_2_S-donor, the 3D samples were lysed according to a here optimized procedure. Five MDA-microspheres were used for each sample, therefore, the expression results can be considered inherently mediated. The treatment with a concentration of 17.0 μM of H_2_S increased the expression of cyclin D1 and the phosphorylation of ERK1/2 in cells grown in 3D culture, as shown in Fig. 6D. These results, together with those of cell viability, "*gel in gel*" assay and L/D assay, suggest that the same concentration of H_2_S that in 2D causes cell death, in the 3D systems has opposite effects increasing the cellular metabolism and migration.

**Fig. 7:**
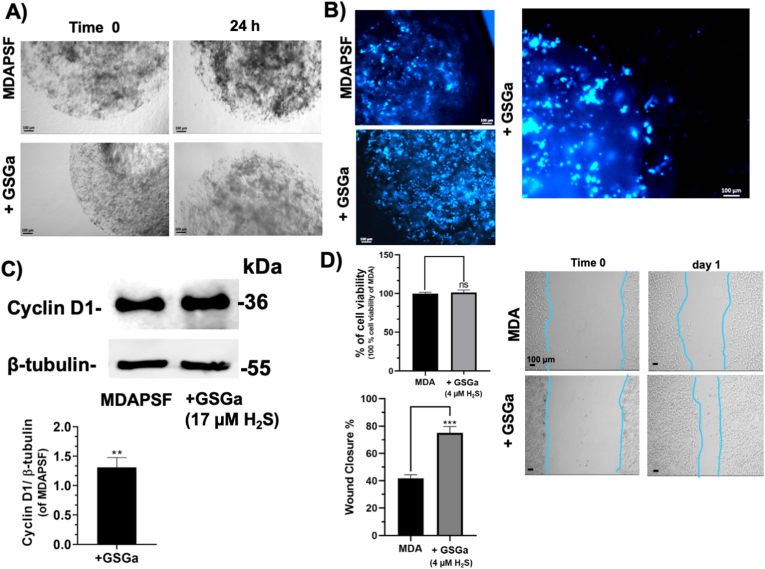
Effects of GSGa on cell migration of MDAPSFs and 2D MDA cell cultures. A) Optical micrographs of **“***gel in gel*” cell invasion assay of MDAPSFs (3 μl) monitored for 24 h of growth in the absence or in the presence of GSGa (releasing 17.0 μM of H_2_S) added in the cell culture medium; **B)** fluorescence micrographs of MDAPSFs (3 μl) in the “*gel in gel”* system after 3 days of growth with and without GSGa (releasing 17.0 μM of H_2_S) using a live-cell fluorescent staining of the nuclei with Hoechst 33342; **C)** cyclin D1 expression in MDAPSFs in the presence and in the absence of GSGa (releasing 17.0 μM of H_2_S); **D)** WST-1 cell viability assay of MDA grown on TCP after 24 h of cell growth without and with GSGa (4.0 μM of H_2_S) in the culture medium and scratch wound healing assay with quantification of wound closure after 24 h of cell growth. The results were obtained by three biological replicas. Error bar indicates S.D. **p value ≤ 0.01, ***p value ≤ 0.0005.

The MDAPSFs in the *“gel in gel”* systems were in better conditions when GSGa was present in the medium at the concentration releasing 17.0 μM of H_2_S as shown in the optical and fluorescence micrographs (Fig. 7A and B). The MDA cells in this system showed the ability to invade the outer gel only in the presence of GSGa (Fig. 7B). In agreement with this result, an increase of the cyclin D1 expression after 24 h of treatment with GSGa was observed. The effect of the GSGa on the cell migration was also assessed in 2D cell culture at the lower concentration, 4.0 μM of H_2_S, which does not affect the cell viability (Fig. 7D). The GSGa treatment at this concentration induced a significant increase of about 40 % of the cell migration, as shown in Fig. 7D. Therefore, it is possible to consider that the concentration of the *gastransmitter* is reduced by the lower diffusion rate in the “*gel in gel”* system that leads to positive antioxidant effects on the breast cancer cells and to an increase of the cell migration, such as observed at low concentration in 2D cell cultures. Therefore, these results clearly show that it is possible to have the opposite effects of the H_2_S donors in 3D systems at the concentration used for 2D systems and suggest the necessity to optimize administration in the 3D cellular models of the H_2_S slow-releasing agents before *in vivo* experiments, in order to not have illicit unintended effects.

### 3.5 Effects of the tumoroid stiffness on the cyclin D1 expression and multi-drug resistance (MDR)

In the past few years, the micro-environment of cancer cells has assumed a very relevant importance in the study of the cancer cell transformation and sensitivity to the drugs [55]. In order to investigate on the molecular basis of the different behavior of the cancer cells to the GSGa treatment in the 3D systems, the effects of the hydrogel stiffness on the MDAPSF system were evaluated, including protein expression and cell migration. In order to simulate the stiffness range of the *in vivo* normal tissue and invasive carcinoma, that was reported to be from 3 to 13 kPa [36], MDAPSFs with different stiffnesses were produced using 2.25, 4.5 and 9 % (w/v) of PEGDa and the cyclin D1 expression was assessed.

**Fig. 8:**
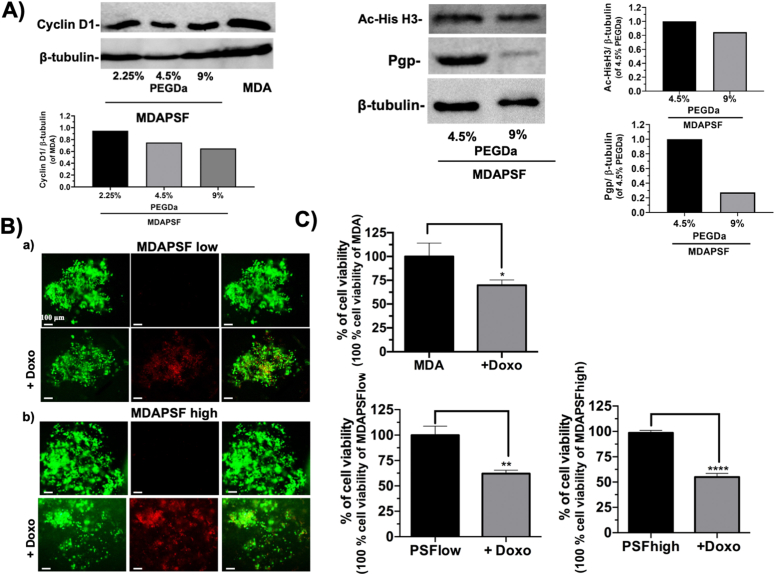
Cell invasion and drug resistance of the breast cancer cells are tunable by isotropic stiffness changes. A) Cyclin D1 expression in MDAPSFs at different stiffness (2.25, 4.5 and 9.0 % of PEGDa) after 48 h of cell growth. The quantitative evaluation of the protein expression is obtained by 5 microspheres for each sample, and is therefore inherently mediated; **B)** L/D assay of MDAPSFs at low (a) and high (b) stiffness after 24 h of treatment with and without 2 μM of doxorubicin, the experiment was performed in triplicate; **C)** cell viability of MDA-MB 231 on TCP, MDAPSFlow and MDAPSFhigh after 24 h of treatment with and without 2 μM of doxorubicin. *p value ≤ 0.05, **p value ≤ 0.01, ****p value ≤ 0.0001.

The cancer cells were analyzed after 24 h of embedding into the PSF for the protein expression and, as shown in Fig. 8A, a down-regulation of the expression of the cyclin D1 was observed in a stiffness-dependent manner, indicating that the expression of this protein and, consequently, the invasiveness of the cancer cells in these systems is linked to the stiffness of the tumoroid. The acetylation of the histone H3 was also reduced by the increase of stiffness. The result is in agreement with the existing link between cyclin D1 and the histone acetylation [56,57].

Moreover, we also investigated the drug sensitivity of the cancer cells in these 3D systems observing an inverse correlation between the Multi-Drug Resistance (MDR) glycoprotein P (Pgp) expression and the stiffness of the tumoroids (Fig. 8A). The down-regulation of the Pgp expression in the MDAPSFs with higher stiffness was also in agreement with the major sensitivity to the doxorubicin with respect to the MDAPSFs with lower stiffness, as shown in Fig. 8C by the Live/Dead assay. In agreement, the metabolic assay performed after only 24 h of treatment with 2 μM of doxorubicin (Fig. 8B) shows that the cancer cells grown in high stiffness (MDAPSFhigh) have a lower percent of cell viability (54.93 % ± 2.03) with respect to their control in the absence of doxorubicin than those observed in the case of MDA grown on TCP (69.89 % ± 3.17) or of MDAPSFlow (62.05 % ± 1.93), 15 % and 7 % less, respectively. Furthermore, a major resistance (about 32 %) to the doxorubicin was also observed at lower stiffness in MDAPF with respect to the MDAPSFlow (see Fig. 3S).

### 3.6 Effects of the stiffness on the *trans*-differentiation of MDA-MB 231 cancer cells into osteoblast-like cells

The observed changes of the cellular morphology, migration and drug-resistance in the MDAPSFs tumoroids, also related to changes in the protein expression, prompted us to investigate the ability of high stiffness of the 3D systems to induce a transformation of the breast cancer cells. Recently, it has been demonstrated the possibility of reprogramming 6 tumor cell lines (KMG4 (brain), HeLa (cervix), A549 (lung), WiDr (colon), J82 (bladder) and Fuji (synovium)) in cancer stem cells (CSCs), culturing them on a double-network (DN) hydrogel made from a copolymer of 2-acrylamide-2-methylpropansulfonic acid and N, N′-dimethylacrylamide [58].

**Fig. 9:**
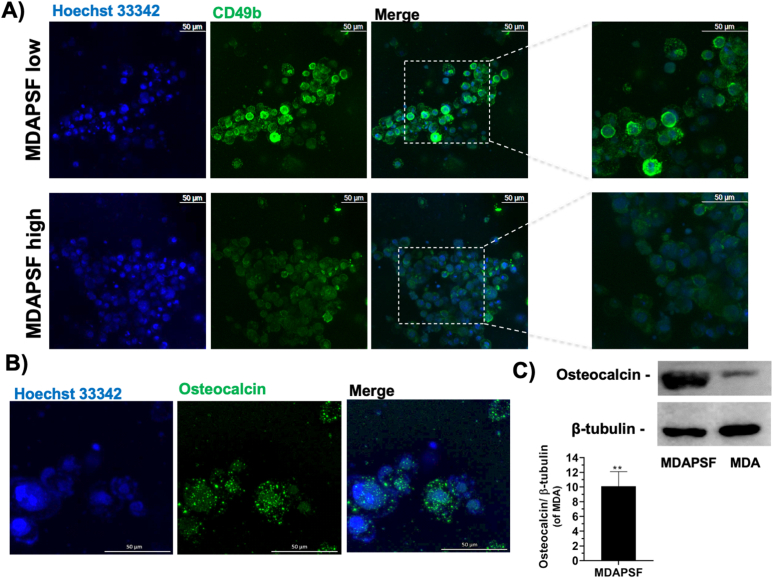
CD49b and osteocalcin expression in MDAPSF. A) Confocal micrographs of MDAPSFlow and MDAPSFHigh after 3 days of cell culture. Cell nuclei are stained using a live-cells fluorescent staining with Hoechst 33342 and CD49b is stained in green (Z-stack by the overlapping of 100 slices); **B)** confocal micrographs of MDAPSFhigh with osteocalcin stained in green (Z-stack by the overlapping of 43 slices) and **C)** osteocalcin expression assessed by western blot analysis of MDAPSFhigh after 3 days of cell growth. Scale bars 50 μm.

To analyze the effects of the stiffness on the MDA-MB 231 cells, we evaluated the expression of the CD49b/integrin α2. Several studies have shown that CD49b is strongly expressed in breast cancer, also in the triple negative MDA-MB 231 cell line and, on the contrary, is weakly present in the background epithelia [59,60]. We found that after only a few days of cell growth in the two systems, MDAPSFhigh and MDAPSFlow, the cancer cells showed a different expression of the CD49b, with an evident down-regulation of the expression in the stiffer 3D system (Fig. 9A). Fig. 9A shows the fluorescence micrographs of the cells stained with AlexaFluor488-Ab-CD49b after three days of cell growth into MDAPSFlow and MDAPSFhigh systems. In the MDAPSFhigh an up-regulation of the osteocalcin expression respect to the cells grown in 2D culture system was also observed. In Fig. 9B are shown the fluorescence micrographs of MDAPSFhigh after three days of cell growth stained with AlexaFluor488-Ab- osteocalcin (OC) and Hoechst, indicating the presence of OC, which was not detectable in MDA-MB 231 cells grown on TCP (data not shown). The significant increase of the expression of OC of about 10-fold in MDAPSFs after three days of cell growth was also confirmed by western blotting analysis, as shown in Fig. 9C.

The down-regulation of CD49b and the increase of OC in MDAPSFs after only three days of cell growth are evidence of a rapid *trans*-differentiation of the MDA-MB 231 cells. Altogether, the cell morphology, the up-regulation of the OC and the positive Alizarin red staining (see Fig. 4S) suggest a *trans*-differentiation of the cancer cells in osteoblast-like cells.

## 4 Discussion

The optimization of 3D cancer models represents an important research field for the production of biologically relevant tools in preclinical drug testing. In this context, 3D tissue analogs should try to replicate the complexity of the biological milieu, including the biophysical and biochemical features of the cellular micro-environment of the tumor. The current 3D models made from collagen or matrigel hydrogels are orders of magnitude softer compared to the stiﬀness of most organs and tumor tissues. Therefore, in the current study we aimed to apply tunable PF and PSF hydrogels in order to more effectively mimic the *in vivo* tumor micro-environment (TME). We produced 3D tumoroids, MDAPFs and MDAPSFs, containing a human breast cancer cell line and we assessed the effects of GSGa, a slow H_2_S releasing extract, and of the common drug doxorubicin using these unique 3D tumor models. Several studies on H_2_S releasing donors have shown that they can induce cell death of cancer cells *in vitro*. GSGa showed a good capability to induce an anti-proliferative effect on MDA-MB 231 cancer cell line in 2D culture, that was comparable to that of GYY4137, which is currently the most relevant slow-H_2_S releasing agent with confirmed *in vivo* anticancer effects [16,61]. In fact, exogenous donors of this *gasotransmitter*, as well as the garlic-derived diallyl sulfide, diallyl disulfide and diallyl trisulfide, have shown anti-tumor properties *in vivo* at high concentrations [62]. Therefore, we speculate that experiments using 2D cultures are of limited value in predicting the *in vivo* efficacy of these H_2_S donors as anti-tumor agents. In our work, the effects of a slow H_2_S*-*donor on breast cancer microspheres were evaluated in a 3D context, resulting in a reverse effect when compared to the 2D cultures at the same concentrations. The presence in the medium of the GSGa also induced a significant increase, about of 40 %, of the cancer cell invasion. This effect was concomitant with an increase of the cyclin D1 expression and ERK1/2 phosphorylation. The concentration of the H_2_S in the 3D culture and “*gel in gel”* system could be reduced by the lower diffusion rate of the fluids in this system, leading to positive antioxidant effects on the breast cancer cells and to an increase in the cellular invasion, such as observed at lower concentration in 2D cell cultures. Therefore, these results clearly suggest the necessity to optimize in the 3D cellular models the administration of the slow H_2_S- releasing agents before *in vivo* experiments in order to avoid the increase of the proliferation and invasion of the cancer cells.

The different cell behaviors to the H_2_S administration could be linked to the change in the 3D isotropic micro-environment. Interestingly, our results show an important link between these effects and the different stiffness of the 3D system. Distinct effects of the GSGa in the stimulation of the invasion were observed in the two 3D systems, MDAPFs and MDAPSFs. These differences were certainly related to the different expression of cyclin D1 in the cells after embedding in the hydrogels. A down-regulation of the expression of cyclin D1 in stiffness-dependent manner was detected, demonstrating that the expression of cyclin D1 could be a marker of sensitiveness of these breast cancer cells to this physical factor.

A hyperactivation of Wnt/β-catenin signaling has often been observed in cancer, this pathway regulates the cyclin D1 expression and histone acetylation and it is linked to the transduction of mechano-physical signals. Therefore, the down-regulation of the cyclin D1 and hypoacetylation of histone H3 could suggest a stiffness-dependent reduction of this pathway activation, driving a *trans*-differentiation process.

In the MDAPSF and MDAPF, the cancer cells showed a globular phenotype with a good cell viability even after several days. The morphological changes might suggest a reversion to the condition of stemness of these cancer cells. Recent studies have shown that tumor cells can be reprogrammed into tumor stem cells (CSCs) capable of differentiating [58]. These studies demonstrated the possibility of reprogramming tumor lines into CSCs by culturing them on a hydrogel matrix [58]. Also in this case the globular phenotype of the cells was observed, and the cells were in the stemness condition showing a high expression of the marker genes Oct3/4, Sox and Nanog. Although herein we did not evaluate the expression of the stemness markers, the MDA-MB 231, as described in the literature [63,64], are poorly differentiated with a characteristic CD44^+^/CD24^-^/low phenotype that has been associated to the CSCs. All the cellular changes that we detected after 24 and 48 h of embedding into the hydrogel at higher stiffness, such as morphology, expression of the cyclin D1, histone H3 acetylation as well as the different sensitivity to H_2_S, which was reported to increase cell viability of the normal or mesenchymal stem cells [20,27,35,65,66], are in agreement with a physically induced *trans*-differentiation (PiT) of the cancer cells.

In the cancer progression relevant roles, in fact, are played by tissue stiﬀness and remodeling of the TME induced by the cancer cells during the tumor growth that promotes the cell invasiveness. Rheological analyses of tissue biopsies of breast tissue including normal tissue, benign tumors, and invasive tumors showed that late, invasive stage tissue has softer regions as a result of the cancer cells inﬁltrating the surrounding tissue [67]. The changes of the ECM stiffness could induce either a transformation of normal mammary epithelium into a malignant phenotype [68] or promote an epithelial to mesenchymal transition (EMT) in pancreatic cancer [69], and have an impact on the chemosensitivity of leukemias [70]. Hence, the cross-talk between cancer cells and their TME plays a pivotal role not only in the progression of the disease, but also in the response to the therapy. Therefore, we also investigated the drug sensitivity of the cancer cells in these 3D systems observing an inverse correlation between the Multi-Drug Resistance (MDR) Pgp expression and the stiffness of the tumoroids. The 5-fold down-regulation of the Pgp expression in the MDAPSFs with higher stiffness was in keeping with the major sensitivity to doxorubicin with respect to the MDAPSFs with lower stiffness. In this context, recently, the relationship between substrate rigidity and drug activity have been investigated using *ex vivo* experiments [[71], [72], [73], [74]]. In *ex vivo* experiments, breast cancer cells cultured in a 2D multi-well hydrogel array on ‘*soft*’ substrates showed a lower response to chemotherapeutics, such as paclitaxel and doxorubicin, as compared to those grown on rigid surfaces, often with multiple orders of magnitude. The doxorubicin cytotoxicity was reduced by 3–10 fold in all the tested cell lines when cultured on the softest substrate versus glass [75]. In agreement with our results, multi-drug resistance proteins of the ATP-binding cassette (ABC) transporter superfamily were highly expressed when primary tumor cells were cultured on soft gels, while these genes are down-regulated in cells seeded onto glass [75]. In our case, after only 24 h of embedding into the PSF hydrogel, the cancer cells show a lower resistance to the doxorubicin, 15 % less of cell viability, than the cells grown on TCP.

Many clinical studies on breast cancer have also demonstrated a change in calcium homeostasis, with the presence of calcifications in tumor bioptic tissues. In breast cancer biopsies the overexpression of several bone matrix proteins, including bone sialo-protein, osteopontin and osteonectin, has been observed. The formation in the breast cancer of Breast Osteoblast-Like Cells (BOLCs), originated from breast epithelium by mesenchymal transformation (EMT) and able to produce microcalcification made of hydroxyapatite (HA), has been described [76]. We have performed our studies using 3D models at a stiffness ranging from 0.3 to 13 kPa (MDAPF, MDAPSFlow and MDAPSFhigh), in agreement with the observed stiffness of freshly isolated breast tumors that are characterized by a primary stiffness regime between 0.1 and 5 kPa and a smaller contribution of rigidities between 5 and 25 kPa [75,77]. In this context, we have demonstrated in our 3D models that a cellular *trans*-differentiation of the MDA-MB 231 can be induced by the single presence of a rheological factor, the stiffness >10 kPa, for a very limited time. In our 3D models with higher stiffness, we observe the osteoblast-like transition of the MDA-MB 231. To investigate the PiT of the cancer cells into MDAPSFhigh system, we have analyzed the protein expression, observing an up-regulation of the osteocalcin after only three days of cell growth. Osteocalcin (OC), or bone γ-carboxyglutamic acid protein, is a protein expressed and secreted by osteoblast cells [78]. It is characterized by three glutamic acid residues that can be carboxylated, whilst the uncarboxylated form of osteocalcin controls physiological pathways in an endocrine manner [79]. A recent study has demonstrated that the uncarboxylated osteocalcin (GluOC) can also play a significant role in promoting the proliferation and metastasis of MDA-MB 231 cells *via* the TGF-β/SMAD3 signaling pathway and, further, lead to an up-regulation of the matrix metalloproteinase MMP-2 [80]. The observed increase of OC, the presence of calcium deposits and the down-regulation of CD49b in MDAPSFs are in agreement with the PiT of the MDA-MB 231 cells into osteoblast-like cells, as reported in several *in vivo* and clinical studies [76,81,82]. Therefore, these results suggest that MDAPSFs can represent good models to simulate the *in vivo* conditions and to better investigate the underlying molecular mechanisms. Moreover, although the cells in MDAPSF exhibit reduced drug-resistance, the production of the osteocalcin and other biochemical factors (*e.g.* Ca^2+^, MMPs, miRNAs) could stimulate the un-transdifferentiated cancer cells to increase their proliferation, production of MMPs, with the reduction of the matrix stiffness, and consequently increase of the cell migration and metastasis.

Techniques for quantifying and visualizing cell migration/invasion have become central in cancer research. Therefore, based on our experience gained on "*gel in gel*" assay [32,33], 3DCM-chip [32] was designed and produced with microfluidic system, capable of easier preparation of the assay with high reproducibility and also allowing the use of smaller quantities of cells and reagents. This 3DCM-chip was used to assess the cell migration/invasion of breast cancer cells in response to GSGa. This system is an innovative “*lab-on-chip*” platform to study the cell migration in a 3D hydrogel-based system, capable of mimicking the ECM. The chip allows the creation of “*gel in gel*” systems also using different types of polymers (thermal and photo-polymerizable) (*e.g*. agarose, gelatin and PF, PSF) performing all the potential analyses shown in our previously published work [33].

## 5 Conclusions

We produced breast cancer cell microspheres with tunable protein-based hydrogels, developing a new method for the preparation and the production of controllable and reproducible tumoroids. We found a strong correlation between the cyclin D1 expression and the stiffness of the 3D cell growth system. PiT of the breast cancer cells toward osteoblast-like phenotype was observed after only three days of cell growth at higher stiffness (13 kPa), with up-regulation of the osteocalcin. These models could help to further clarify the molecular bases of the cell micro-environment cross-talk that are not yet well defined. Therefore, the PiT could play a relevant role in the cancer therapy not only to model the disease in high-throughput strategies of drug-screening and to better *in vitro* recapitulate drug-response that may occur in native tumor environments, but also, in case of some types of tumors, by the endoscopically administration of tunable photopolymerizable hydrogels to sensibilize the cancer cells before the chemotherapy and the surgical removal of the tumor.

These results highlight the relevance of integrating a biomimetic physical micro-environment into any *in vitro* model and demonstrate that the stiffness of the tumor micro-environment has relevant effects on the response of breast cancer cells to anticancer therapy.

## Credit author statement

Silvia Buonvino: Methodology, Investigation, Data curation, Writing- Reviewing and Editing; Ilaria Arciero: Investigation, Data curation, Reviewing and Editing; Eugenio Martinelli: Methodology, Reviewing; Dror Seliktar: Methodology, Data curation, Reviewing and Editing; Sonia Melino: Conceptualization, Visualization, Investigation, Methodology, Data curation, Supervision, Writing- Original draft preparation, Writing- Reviewing and Editing.

## Funding

The research leading to these results has received funding from the European Union – Next Generation EU through the Italian Ministry of University and Research under PNRR - M4C2-I1.3 Project PE_00000019 "HEAL ITALIA”. The views and opinions expressed are those of the authors only and do not necessarily reflect those of the European Union or the European Commission. Neither the European Union nor the European Commission can be held responsible for them. This research has been also partially supported by 10.13039/501100007642University of Rome Tor Vergata funding to TuCaDi project.

## Declaration of competing interest

The authors declare that they have no known competing financial interests or personal relationships that could have appeared to influence the work reported in this paper.

## Data Availability

Data will be made available on request.
