# Supplementary material for: Modelling the disease: H2S-sensitivity and drug-resistance of triple negative breast cancer cells can be modulated by embedding in isotropic micro-environment
Source: Mater Today Bio. 2023 Nov 11;23:100862. doi: 10.1016/j.mtbio.2023.100862 (PMC10689286; doi:10.1016/j.mtbio.2023.100862)
Supplement: Multimedia component 1 [file mmc1.docx]

**Supplementary Materials**


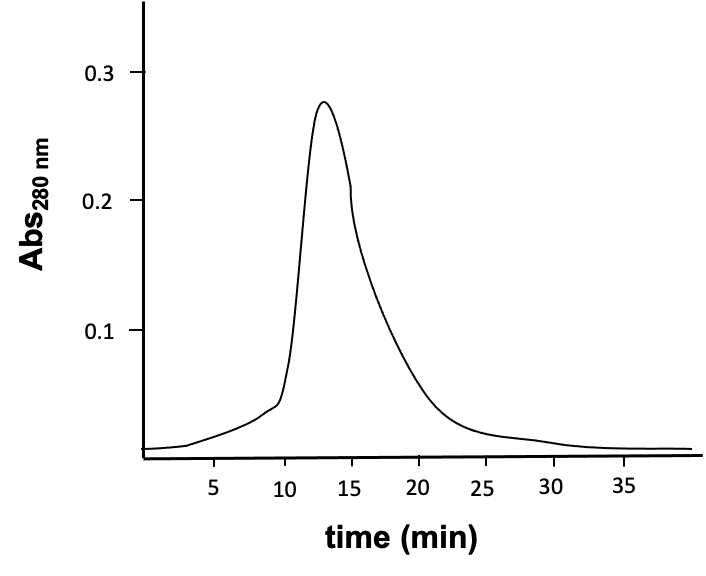


**Fig.1S**  Gel filtration of the Fibroin extract from *Bombix mori* cocoon and used for the preparation of the PEG-silk fibroin hydrogel (PSF)

**PDMS**

**B)**

**A)**


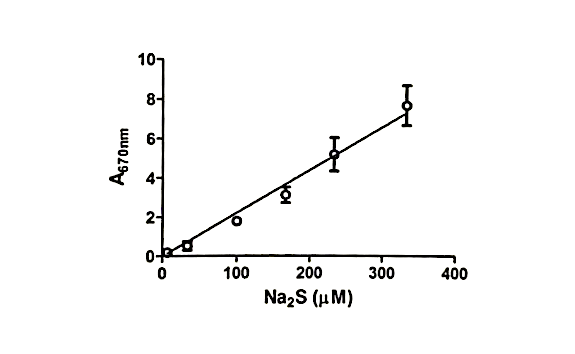

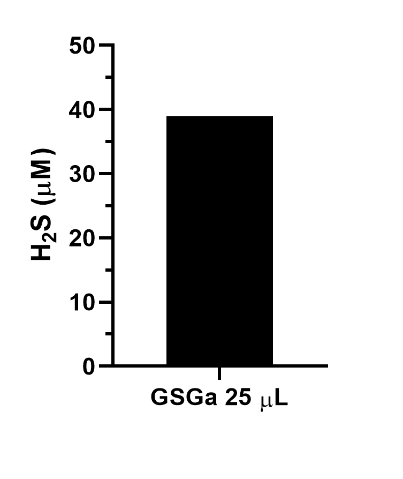


**Fig.2S** A) Calibration curve of the H_2_S-releasing by methylene blue assay (MB assay) obtaining using different concentrations of Na_2_S solution; B) H_2_S- release of 25 µl of GSGa by MB assay.

**B)**

**A)**

**
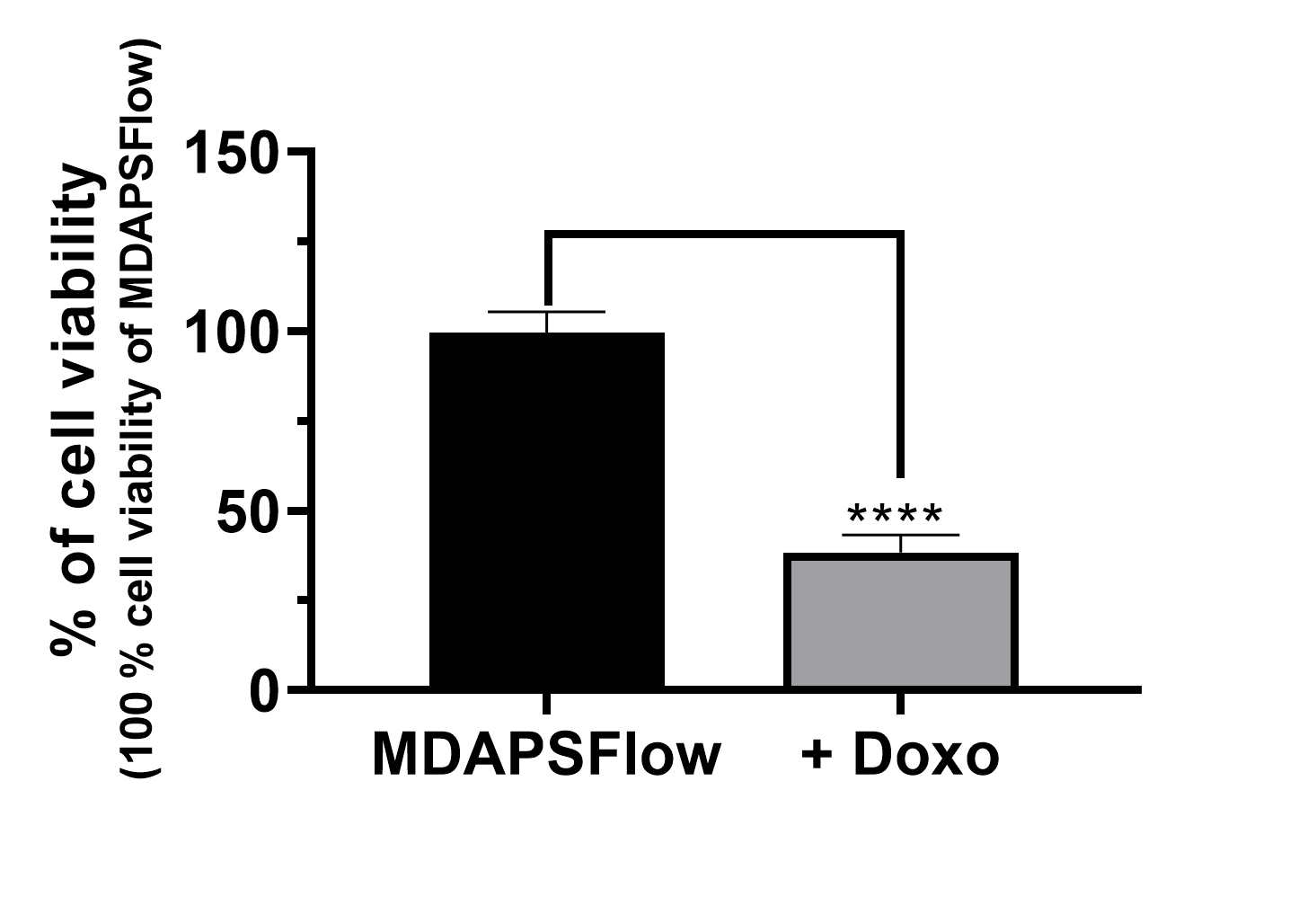
**

**
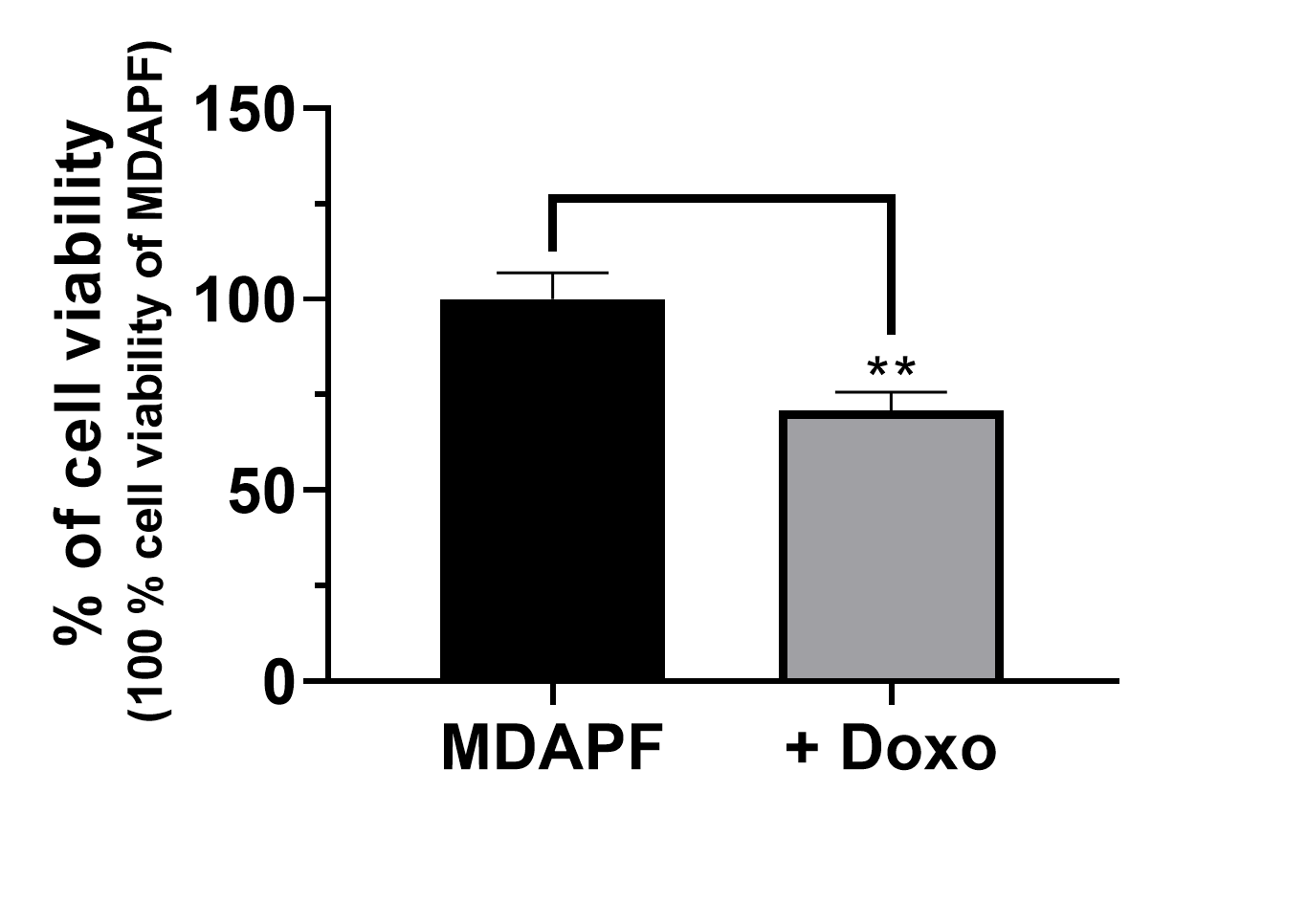
**


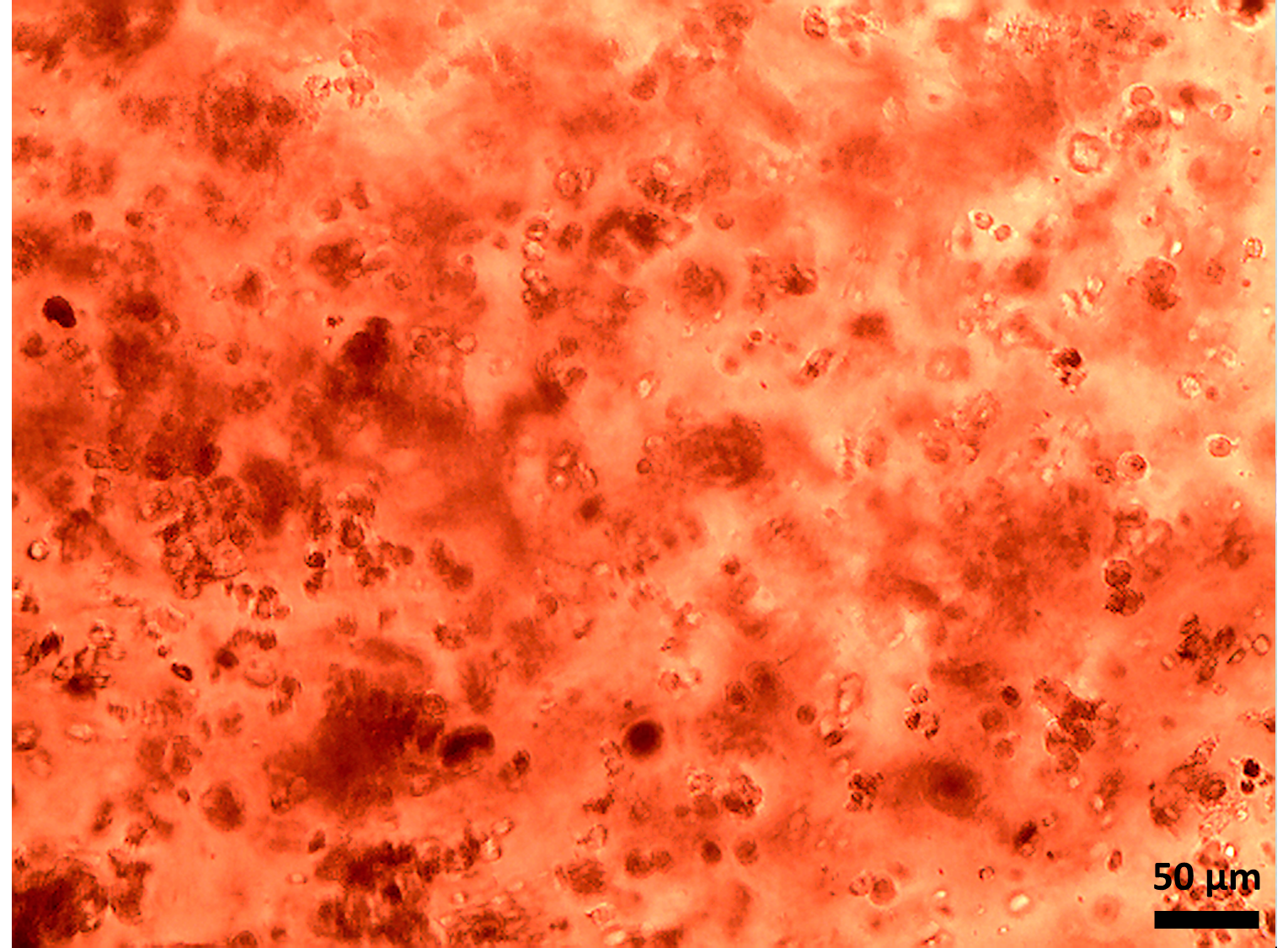
**Fig. 3S** Cell viability of MDAPF (A) and MDAPSFlow (B) (4000 cells/µl) after 24 h of treatment with 2 µM doxorubicin. ** p value < 0.002 **** p value < 0.0001.


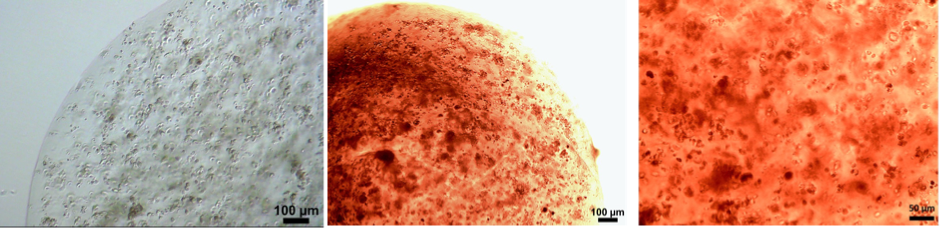


**Fig. 4S** Optical Micrographs of MDAPSFhigh spheres after three days of cell growth before and after Alizarin red staining. Scale bars 100 and 50 µm.
